# Supplementary figures and images for: An Overview on MADS Box Members in Plants: A Meta-Review
Source: Int J Mol Sci. 2024 Jul 28;25(15):8233. doi: 10.3390/ijms25158233 (PMC11311456; doi:10.3390/ijms25158233)

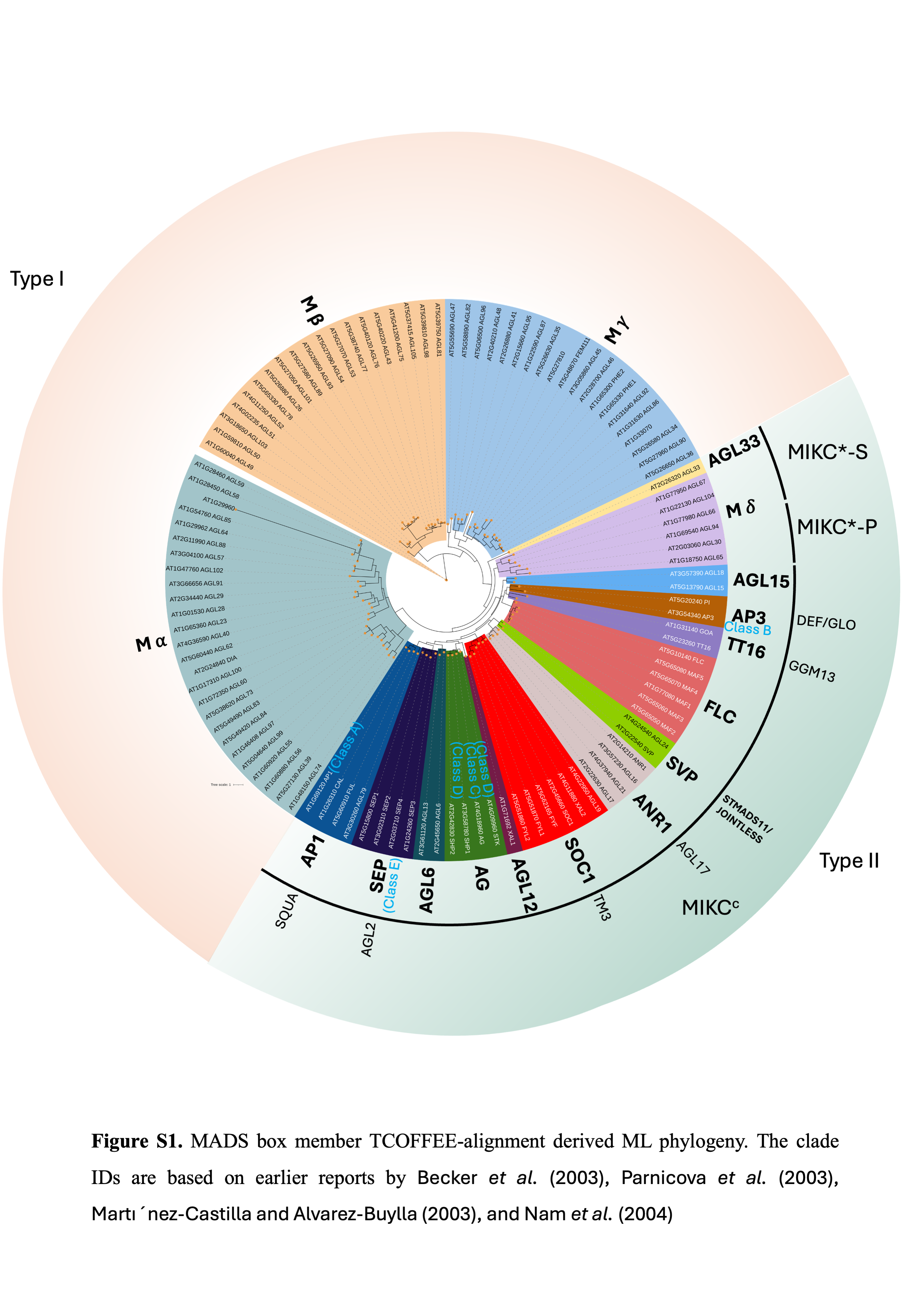

Supplement: Supplementary file 1 [file ijms-25-08233-s001.zip › Supplementary Data/Figure S1.png]

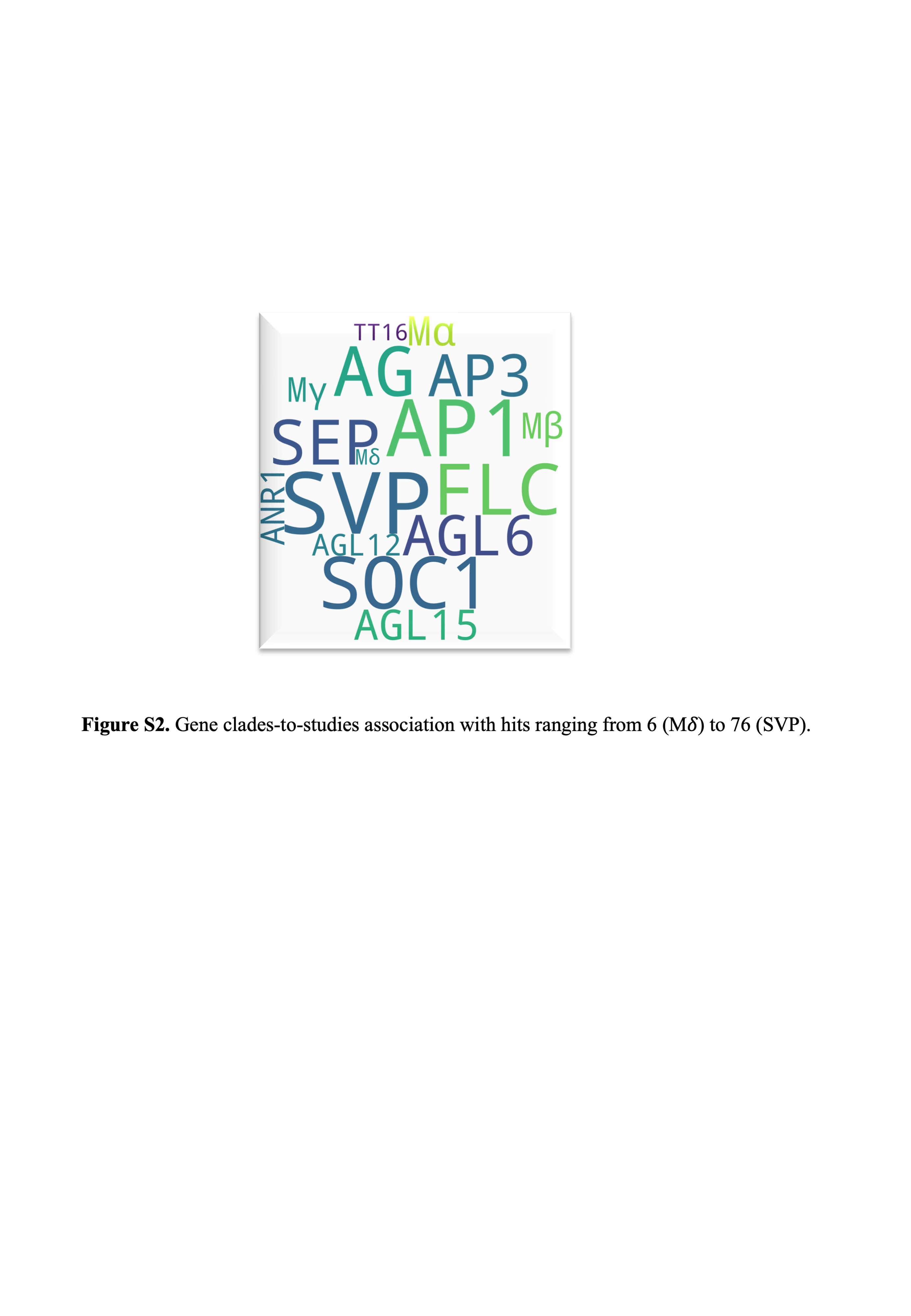

Supplement: Supplementary file 1 [file ijms-25-08233-s001.zip › Supplementary Data/Figure S2.png]
